# User Engagement and Attrition in an App-Based Physical Activity Intervention: Secondary Analysis of a Randomized Controlled Trial

**DOI:** 10.2196/14645

**Published:** 2019-11-27

**Authors:** Sarah Edney, Jillian C Ryan, Tim Olds, Courtney Monroe, François Fraysse, Corneel Vandelanotte, Ronald Plotnikoff, Rachel Curtis, Carol Maher

**Affiliations:** 1 Alliance for Research in Exercise, Nutrition and Activity University of South Australia Adelaide Australia; 2 Health and Biosecurity Commonwealth Scientific and Industrial Research Organisation Adelaide Australia; 3 Arnold School of Public Health University of South Carolina Columbia, SC United States; 4 Physical Activity Research Group, Appleton Institute, School of Health, Medical and Applied Sciences Central Queensland University Rockhampton Australia; 5 Priority Research Centre for Physical Activity and Nutrition The University of Newcastle Newcastle Australia

**Keywords:** physical activity, smartphone, behavior

## Abstract

**Background:**

The success of a mobile phone app in changing health behavior is thought to be contingent on engagement, commonly operationalized as frequency of use.

**Objective:**

This subgroup analysis of the 2 intervention arms from a 3-group randomized controlled trial aimed to examine user engagement with a 100-day physical activity intervention delivered via an app. Rates of engagement, associations between user characteristics and engagement, and whether engagement was related to intervention efficacy were examined.

**Methods:**

Engagement was captured in a real-time log of interactions by users randomized to either a gamified (n=141) or nongamified version of the same app (n=160). Physical activity was assessed via accelerometry and self-report at baseline and 3-month follow-up. Survival analysis was used to assess time to nonuse attrition. Mixed models examined associations between user characteristics and engagement (total app use). Characteristics of super users (top quartile of users) and regular users (lowest 3 quartiles) were compared using *t* tests and a chi-square analysis. Linear mixed models were used to assess whether being a super user was related to change in physical activity over time.

**Results:**

Engagement was high. Attrition (30 days of nonuse) occurred in 32% and 39% of the gamified and basic groups, respectively, with no significant between-group differences in time to attrition (*P*=.17). Users with a body mass index (BMI) in the healthy range had higher total app use (mean 230.5, 95% CI 190.6-270.5; *F*_2_=8.67; *P*<.001), compared with users whose BMI was overweight or obese (mean 170.6, 95% CI 139.5-201.6; mean 132.9, 95% CI 104.8-161.0). Older users had higher total app use (mean 200.4, 95% CI 171.9-228.9; *F*_1_=6.385; *P*=.01) than younger users (mean 155.6, 95% CI 128.5-182.6). Super users were 4.6 years older (t_297_=3.6; *P*<.001) and less likely to have a BMI in the obese range (χ^2^_2_=15.1; *P*<.001). At the 3-month follow-up, super users were completing 28.2 (95% CI 9.4-46.9) more minutes of objectively measured physical activity than regular users (*F*_1,272_=4.76; *P*=.03).

**Conclusions:**

Total app use was high across the 100-day intervention period, and the inclusion of gamified features enhanced engagement. Participants who engaged the most saw significantly greater increases to their objectively measured physical activity over time, supporting the theory that intervention exposure is linked to efficacy. Further research is needed to determine whether these findings are replicated in other app-based interventions, including those experimentally evaluating engagement and those conducted in real-world settings.

**Trial Registration:**

Australian New Zealand Clinical Trials Registry ACTRN12617000113358; https://www.anzctr.org.au/ACTRN12617000113358.aspx

## Introduction

### Background

Mobile phone apps have been proposed as a cost-effective method of delivering wide-scale, appealing interventions, targeting lifestyle-related health problems, such as physical inactivity, to prevent chronic diseases [1,2]. However, despite the rapid growth of these approaches, researchers only started to develop and examine custom-made apps to deliver physical activity (PA) interventions in the past decade [3]. To date, these have tended to report mixed results [4,5], with meta-analyses indicating overall modest effects [6,7]. Contributing to overall modest efficacy are the lower rates of intervention exposure and compliance and the high levels of attrition that are often reported [8].

The apparent link between intervention exposure and efficacy [8-10] highlights the need for detailed understanding of user engagement, which considers how frequently users access or use different features of the app or simply log on to the app at all. To date, engagement has been conceptualized and operationalized in different ways, and attempts have been made to reach a shared understanding of approaches [11,12]. Research studies have tended to focus on frequency of app use and dropout rates assessed as percentages of users who cease using the app during the intervention [12,13]. Others take a user-centric approach, considering the appeal of an app and the experience of using it reported directly from users themselves, via interviews or questionnaires [14]. Commercial companies, on the other hand, view app engagement differently, focusing on metrics that include the number of daily active users (DAUs), the number of monthly active users (MAUs), and *churn*, measured either as the rate at which nonuse attrition occurs or, to take into account new app downloads, by dividing the number of users at the end of a period by the number of users present at the beginning and expressing this as a percentage [15]. Finally, commercial companies examine the characteristics and usage patterns of *super users*, who use an app the most in terms of frequency, time spent, or who remain active users for an extended period. These super users are more likely to rate an app favorably, share it with their friends, and engage with most of its features [16-19]. More recently, as researchers look to disseminate apps with demonstrated efficacy into natural, ecologically valid settings [20-22], it may be more useful to adopt these commercial metrics. In this approach, engagement metrics used to measure the success of for-profit commercial apps may be more informative or provide unique insights and could be adopted to allow for comparison between research- and industry-led PA apps.

### Objectives

This study therefore sought to contribute to addressing this need by applying engagement metrics from commercial settings to app usage data collected within a health behavior randomized controlled trial (RCT) to provide insights regarding usage rates and associations between user characteristics and app usage.

This study aimed to (1) describe user engagement with a PA app as total app use, DAU and MAU, and nonuse attrition (*churn rate*), (2) examine whether user characteristics were associated with engagement (total app use), (3) compare characteristics between *super users* and *regular users*, and (4) examine whether total app use or being a super user was related to intervention efficacy.

## Methods

### Research Design

This study is a secondary and subgroup analysis of the 2 intervention arms from a 3-group cluster RCT that evaluated the efficacy of an app-based intervention in increasing performance of moderate-to-vigorous PA (MVPA), in comparison with a waitlist control group. Full details of the RCT have been published previously [23]. The study received ethics approval from the University of South Australia’s Human Research Ethics Committee (protocol: 33967), and it was registered with the Australian and New Zealand Clinical Trial Registry (protocol: 12617000113358).

### Participants and Procedures

Recruitment took place in 2016 and 2017, primarily via a paid Facebook advertising campaign and free advertisements placed in community-based Facebook groups. Participants were eligible if they were aged 18 to 65 years, lived anywhere in Australia, used Facebook weekly, reported as completing less than 150 min of MVPA per week, and were able to sign up to the study in a group of at least 3 and a maximum of 8 existing Facebook friends who also met the study inclusion criteria and were willing to join the study.

Participant teams were randomized to either the gamified or basic app (descriptions below) or to the waitlist control group in a 1:1:1 allocation ratio. The gamified app included social and gamified features designed to encourage use of the app and in-app interaction within teams of friends using the app together, whereas the basic app was designed to be used by each participant independently. Participant teams allocated to the waitlist control group are not included in the current analysis.

### Intervention

Users in both app groups received a pedometer (Zencro, TW64S) to measure their daily step counts**,** and they received a weekly email with a summary of their individual step count progress. For gamified users, the email also highlighted a different app feature each week.

#### Gamified App

**Figure 1:**
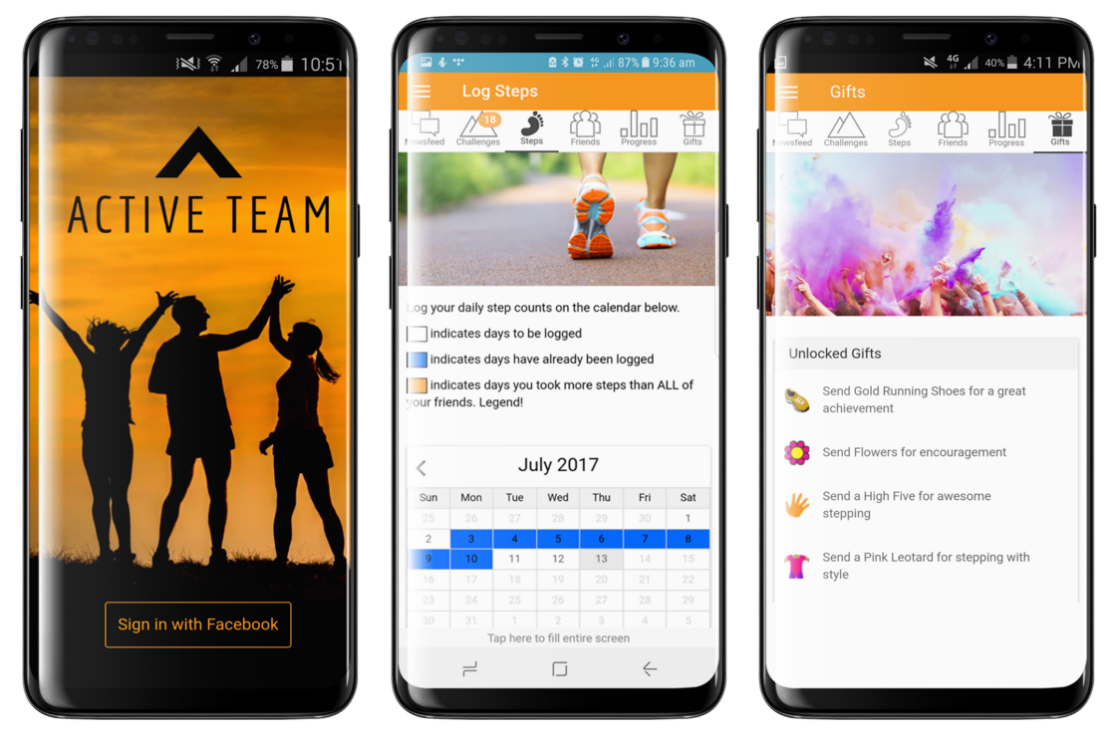
Screenshots of the Active Team app Showing (left to right): splashscreen, step calendar, and virtual gifts.

The gamified app (*Active Team*, see Figure 1) encouraged users to take 10,000 steps per day for 100 days. This app was designed by the research team (led by CM) and developed in conjunction with a software development company (Portal Australia) primarily for academic research purposes. Figure 1 shows screenshots of the app. Full app details and additional images are available in the study protocol [23]. Within the app, users could log their steps into a calendar that tracked their progress over time. Simple gamification (game-like elements included in nongame settings [24]) features were included: a leaderboard, unlockable gifts and medals, a Facebook-style newsfeed, and mini-PA challenges. The leaderboard displayed a ranked summary of each teammates’ step count progress. New virtual gifts and medals were unlocked when users reached predetermined step counts. The Facebook-style newsfeed allowed users to share messages with each other, and mini challenges could be sent between users to encourage short bursts of PA (eg, to take 2000 steps in the next 20 min or to take 12,000 steps per day for 3 consecutive days). Users received a daily push notification reminding them to log their step counts, and users received push notifications when a teammate interacted with them within the app.

#### Basic App

Users of the basic app received a pared down version of the Active Team app that allowed them to enter and monitor their own steps in the calendar, and they received a daily push notification reminding them to enter their step counts. This version of the app contained no gamified or social features.

### Outcomes

This study used demographic and PA data, as well as app usage data from the 100-day intervention period.

#### Demographics

Demographic characteristics were collected within a Web-based survey at baseline. Users reported their sex (male and female), age—median=41 and categorized into older (>41) or younger (<40)—the highest level of education they had achieved (high school or less, some college or further education institution, and university degree or higher), and height and weight, from which body mass index (BMI; kg/m^2^) was calculated and converted into categories: healthy weight (<24.9), overweight (25-29.9), obese (>30).

#### Physical Activity

PA was assessed at baseline (before being randomized to the gamified or basic app) and 3-month follow-up (to coincide with the end of the 100-day intervention period), using GENEActiv accelerometer. At both time points, participants received an accelerometer, instructions to wear the device for 7 days, and a reply-paid envelope, via the Australian postal system. These data were classified into MVPA by using cut points established by Esliger et al [25]. Self-reported PA was collected via the Active Australia Survey (AAS) [26], delivered electronically. The AAS collects details of minutes spent in walking and moderate and vigorous activity in the past week and calculates MVPA from moderate and vigorous activity.

#### Engagement

Engagement data were collected using a real-time log of each user’s interactions with the app, which was automatically uploaded to the study’s server over the 100-day intervention period. The operationalization of the engagement metrics used in this study is provided below.

#### Total App Use

The primary engagement metric used in this study is the number of times the app features were used (*total app use*) during the 100-day intervention period. To calculate this individual-level engagement metric, each interaction with a feature of the app (ie, step calendar, newsfeed, challenge page, gift page, and friends page) was included as a single use. Where the server recorded multiple interactions with a single app feature within a 15-min period, this was counted once only.

#### Daily Active Users

This sample-level engagement metric was calculated as the number and percentage of gamified and basic app users who accessed the app each day.

**Monthly Active Users**

This sample-level engagement metric was calculated as the number and percentage of gamified and basic app users who accessed the app at least once every 30 days.

#### Nonuse Attrition

Attrition was defined as occurring once the user ceased accessing the app for 30 consecutive days or more; the nonuse attrition rate is a sample-level engagement metric. The 30-day threshold is often used to describe the use of commercial apps [15,27]. A sensitivity analysis was performed using the threshold of 14 days of nonuse, a time frame, which has been previously applied in research studies [22,28,29].

#### Superusers

Super users were defined as users whose total app use fell into the top quartile of all users (individual-level engagement metric).

#### Step Calendar Use

The number of unique visits to the step calendar (*step calendar use*) was also calculated as an individual-level engagement metric, and this has been presented descriptively.

DAU, MAU, nonuse attrition, and super users of the step calendar were calculated, as users of both apps could access this feature, whereas users of the gamified app had access to additional gamified and social features, which could potentially increase their total app use (and were designed to achieve this). The results from the 2 metrics (total app use and step calendar use) were near identical; therefore, the results pertaining to step calendar use are provided in Multimedia Appendix 1.

### Statistical Analysis

Demographic characteristics, baseline PA levels, total app use, DAU, and MAU are presented descriptively. Kaplan-Meier survival curves [30] were used to assess the time at which attrition (30 consecutive days of nonuse; sensitivity analysis: 14 consecutive days of nonuse) from each app occurred. The number of days of app use was the time variable, and the event variable was specified as being when the user ceased using the app for 30 or more consecutive days (or 14 or more consecutive days for the sensitivity analysis). Observations were classified as censored when the app was still being used by the end of the 100-day intervention period. The log-rank test was used to determine whether the time until nonuse attrition occurred was statistically significantly different between groups (gamified or basic app group).

Associations between user characteristics (sex, education, BMI, age, and group [gamified or basic app] and engagement [total app use]) were examined using the general linear model procedure. Significant interactions for categorical variables (education and BMI) were followed up with post hoc pairwise comparisons (Kruskal-Wallis tests with Bonferroni correction), where appropriate. Demographic characteristics and patterns of app usage by super users (top quartile of users) and regular users (lowest 3 quartiles) are presented descriptively. Between-group differences were analyzed using independent *t* tests for continuous variables (age, objective, and self-reported PA) and chi-square tests for categorical and binary variables (education, BMI category, and sex). Linear mixed models were used to examine whether total app use or being a super user was related to changes in objective or self-reported PA over time. Total app use, time, and a total app use-by-time interaction were entered as fixed effects in the first model, and superuser status (ie, yes or no), time, and a superuser status-by-time interaction were entered as the fixed effects in the second model. In both models, individual and team were entered as random effects. Adjustments were made for demographic characteristics (sex, education, BMI, and age) by entering these variables into the model. There were no substantial differences between the adjusted and unadjusted models, and the unadjusted models are presented. All analyses were undertaken in SPSS version 25 (IBM Corp).

## Results

### User Characteristics

A total of 301 users were randomized to either the gamified (n=141) or basic (n=160) version of the app. Users in both groups were predominantly female (73.8%, 222/301), university educated (53.2%, 159/301), overweight or obese (35.8%, 107/301 and 43.1%, 129/301), and with an average age of 42 (SD 12) years. There were no statistically significant differences in baseline characteristics between the 2 groups. Overall use of both apps was high. On an average, total app use for the gamified group was 239 (SD 202) compared with 120 (SD 94) for basic app users. Users accessed the step calendar on an average of 44 (SD 32) and 36 (SD 29) times for the gamified and basic apps, respectively. Full details of user characteristics and app usage are presented in Table 1.

**Table 1:** Participant baseline characteristics, total app use, and total step calendar use.

Participant characteristic		Gamified (n=141)	Basic (n=160)	All (N=301)
**Sex, n (%)**				
	Female	106 (75.2)	116 (72.5)	222 (73.8)
**Education level, n (%)**				
	High school or less	26 (18.7)	25 (15.6)	51 (17.1)
	Some college	36 (25.9)	53 (33.1)	89 (29.8)
	University degree	77 (55.4)	82 (51.3)	159 (53.2)
**Body mass index, n (%)**				
	Healthy weight	26 (18.7)	37 (23.1)	63 (21.1)
	Overweight	52 (37.4)	55 (34.4)	107 (35.8)
	Obese	61 (43.9)	68 (42.5)	129 (43.1)
Age (years), mean (SD)		43.3 (11.5)	40.4 (12.2)	41.8 (11.9)
Objective MVPA^a^ minute per day, mean (SD)		106.7 (52.9)	102.7 (50.6)	104.6 (51.6)
Self-reported MVPA minute per week, mean (SD)		243.6 (211.7)	262.1 (264.2)	253.5 (240.9)
Total app use, mean (SD)		239.0 (202.0)	120.17 (94.3)	175.3 (165.2)
Step calendar use, mean (SD)		43.6 (32.4)	36.1 (28.5)	39.5 (30.6)

^a^MVPA*:* moderate-to-vigorous physical activity.

### Daily Active Users

**Figure 2:**
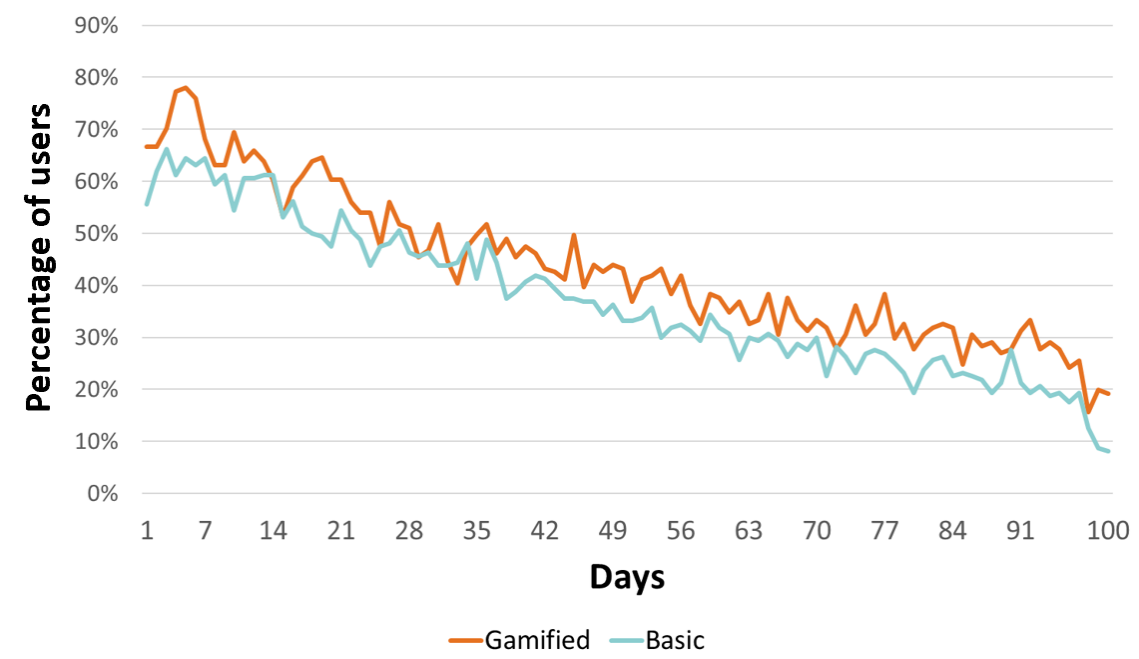
Daily active users and total app use.

Figure 2 shows the sample-level engagement metric of number of users accessing the app each day. The number of DAUs declined over the 100-day intervention period. At day 7, 68.0% (96/141) of the gamified and 64.4% (102/160) of the basic group were accessing the app daily. By day 49, this declined to 43.9% (62/141) of the gamified and 36.2% (58/160) of the basic group. By day 91, 31.2% (44/141) of the gamified and 21.2% (34/160) of the basic group were accessing the app daily. Differences between groups were statistically significant (t=14.96, *P*<.001), favoring the gamified group.

### Monthly Active Users

The sample-level engagement metric, number of MAUs, remained high across the 3-month intervention period. In the first 30 days, 93.7% (282/301) of all users accessed the app (94.3%, 133/141) and 93.1% (149/160) of the gamified and basic groups, respectively). This number declined slightly across the intervention period, but it remained high at 76.4% (230/301) for both groups (75.9%, 107/141) and 76.9% (123/160) of the gamified and basic groups, respectively). Differences in MAUs between the 2 groups were not statistically significant (*P*=.99).

### Nonuse Attrition

**Figure 3:**
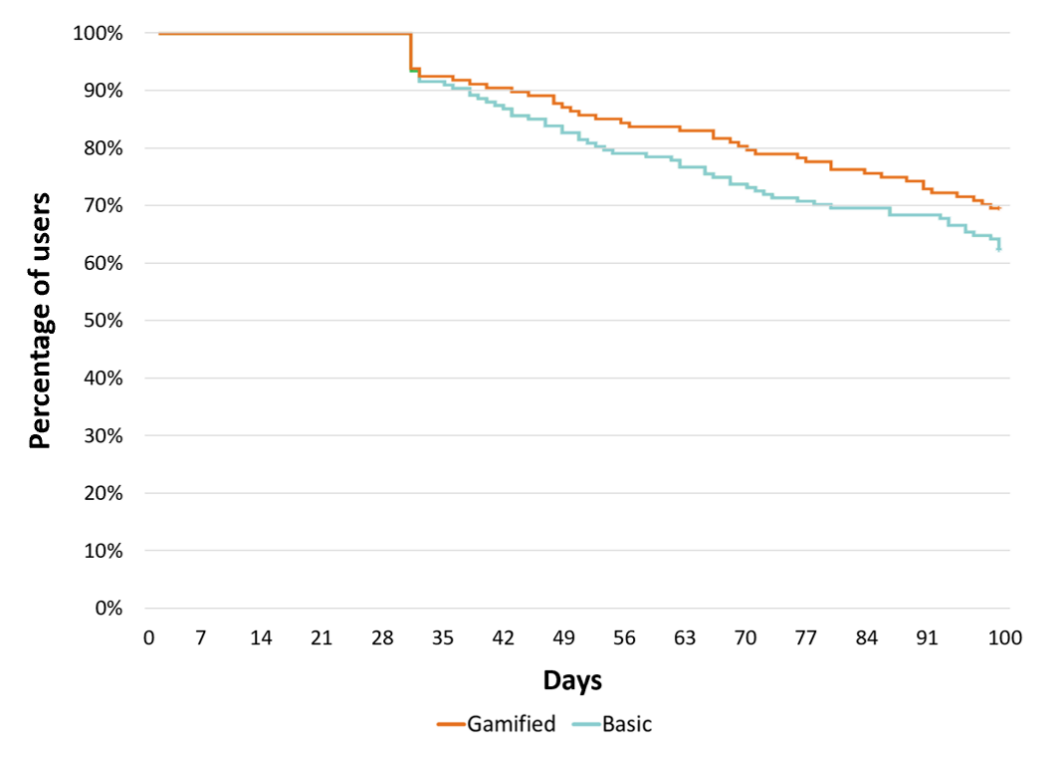
Kaplan-Meier survival estimates showing time to nonuse attrition, 30-day nonuse threshold, and total app use.

**Figure 4:**
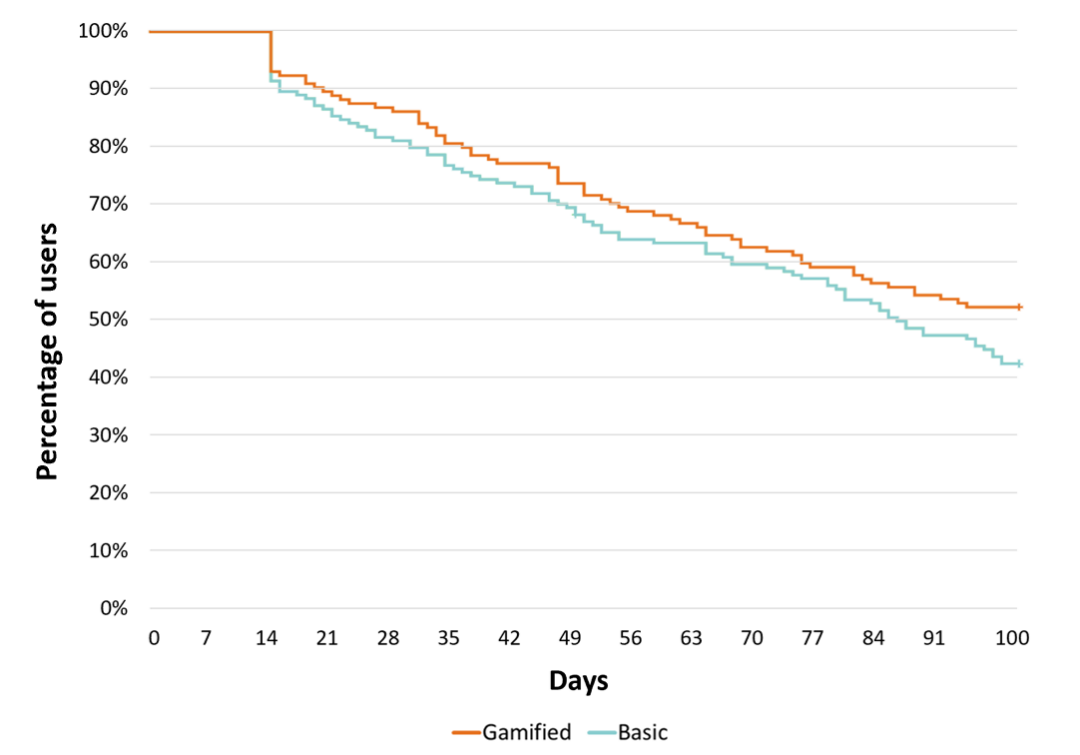
Kaplan-Meier survival estimates showing time to non-use attrition 14-day non-use threshold - total app use.

When assessed using the 30 days of nonuse threshold, nonuse attrition occurred for 31.9% (96/141) and 39.4% (97/160) of the gamified and basic groups, respectively, with no significant differences between groups in the time to attrition (log-rank test, *P*=.17; see Figure 3). Sensitivity analysis applied the 14 days of nonuse threshold and found that nonuse attrition occurred for 48.9% (69/141) and 58.7% (94/160) of the gamified and basic groups, respectively, and differences were not statistically significant (log-rank test, *P*=.12; see Figure 4).

### User Characteristics and Total App Use

Associations between user characteristics and total app use (individual-level engagement metric) were examined using general linear models. Results indicated that BMI, age, and app group (gamified or basic) were each associated with total app use. Post hoc pairwise comparison (Kruskal-Wallis test) indicated total app use varied on the basis of BMI (χ^2^_2_=12.8, *P*=.001), driven by differences between those with a BMI classified as healthy (median total app use=194), compared with obese (median total app use=112; *P=*.001). Older users had higher total app use (mean 200.4, 95% CI 171.9-228.9; *F*_1_=6.39; *P=*.01) than younger users (mean=155.6, 95% CI 128.5-182.6). Users of the gamified app also had more total app use (mean= 236.5, 95% CI 207.9-265.1; *F*_1_=45.12; *P*<.001), when compared with those using the basic app (mean=119.5, 95% CI 93.0-146.0). Full details of all associations are presented in Table 2.

**Table 2:** Associations between user characteristics and total app use.

Characteristics		Total, n	Total app use		
			Mean (95% CI)	*F* test *(df)*	*P* value
**Sex**					
	Male	78	163.7 (129.1-198.2)	2.10 *(1)*	.15
	Female	221	192.3 (170.1-214.6)	—^a^	—
**Education level**					
	High school or less	51	170.8 (128.7-212.9)	2.21 *(2)*	.11
	Some college	89	162.0 (128.0-195.0)	—	—
	University degree	159	201.2 (174.9-227.5)	—	—
**Body mass index**					
	Healthy weight	63	230.5 (190.6-270.5)	8.67 (2)	*<.001* ^b^
	Overweight	107	170.6 (139.5-201.6)	—	—
	Obese	129	132.9 (104.8-161.0)	—	—
**Age (years)^c^**					
	Younger	149	155.6 (128.5-182.6)	6.39 *(1)*	*.01*
	Older	150	200.4 (171.9-228.9)	—	—
**Group**					
	Gamified	141	236.5 (207.9-265.1)	45.12 *(1)*	*<.001*
	Basic	160	119.5 (93.0-146.0)	—	—

^a^Not applicable.

^b^*Italics* indicates statistical significance.

^c^Age: younger=≤40; older=≥41.

### Super Versus Regular Users

Super users used the app an average of 401 (SD 160; range 248 to 1062) times compared with 100 (SD 71; range 0 to 239) times for regular users. Super users were more likely to be a gamified rather than basic app user (χ^2^_1_=29.4; *P*<.001), were 4.6 years older (t_297_=3.6; *P*<.001), and were more likely to have a BMI that placed them within the healthy or overweight, rather than obese, range (χ^2^_1_=15.1; *P*<.001), when compared with regular users.

There were no significant differences between super and regular users for either objectively measured or self-reported PA data at baseline; however, super users had greater increases in their objectively measured PA change scores (t_239_=4.1; *P*<.001), when compared with regular users whose PA decreased slightly from baseline. Full details of differences between super and regular users with regard to baseline characteristics and PA change over time can be found in Table 3.

**Table 3:** Baseline characteristic and physical activity change score comparisons between super and regular users.

Characteristics		Total app use		
		Super	Regular	*P* value
**Sex, n (%)**				
	Male	15 (19.7)	64 (28.4)	.14
	Female	61 (80.3)	161 (71.6)	—^a^
**Education level, n (%)**				
	High school or less	9 (12.0)	42 (18.8)	.09
	Some college	18 (24.0)	71 (31.7)	—
	University degree	48 (64.0)	111 (49.6)	—
**Body mass index, n (%)**				
	Healthy weight	22 (29.3)	41 (18.3)	*<.001* ^b^
	Overweight	35 (46.7)	72 (32.1)	—
	Obese	18 (24.0)	111 (49.6)	—
Age (years), mean (SD)		46.0 (11.5)	40.4 (11.8)	*<.001*
**Group, n (%)**				
	Gamified	56 (73.7)	85 (37.8)	*<.001*
	Basic	20 (26.3)	140 (62.2)	—
Objective PA^c^ minute per day at baseline, mean (SD)		107.0 (45.7)	103.7 (53.6)	.63
Self-reported PA minute per week at baseline, mean (SD)		244.0 (211.2)	256.7 (250.5)	.69
Objective PA minute per day change score, mean (SD)^d^		18.4 (45.0)	–8.1 (47.1)	*<.001*
Self-reported PA minute per week change score, mean (SD)^d^		239.7 (424.4)	157.3 (388.0)	.14

^a^Not applicable.

^b^*Italics* indicates statistical significance.

^c^PA: physical activity.

^d^Physical activity change scores from baseline to 3-month follow-up (end of intervention).

### Total App Use and Changes to Physical Activity

There was a weak, significant total app use-by-time interaction effect for objective PA (*F*_1,272_=4.5; *P*=.04) and self-reported PA (*F*_1,304_*=*6.56; *P*=.01), where higher total app use (individual-level engagement metric) was associated with greater increases in PA at 3-month follow-up.

### Super Users and Changes to Physical Activity

At the 3-month follow-up, objective MVPA had increased from baseline for super users of the app, whereas it decreased slightly for regular users. There was a significant group by time interaction, where super users were completing 28.2 (SE 9.5, 95% CI 9.4-46.9) more min of MVPA than regular users (*F*_1,272_=4.76; *P=*.03). Differences between super and regular users for self-reported MVPA favored the super users (mean 89.7, SE 43.4, 95% CI 4.4-175.1); however, did not reach statistical significance (*F*_1,297_*=*3.31; *P=*.07).

## Discussion

###  Principlal Findings

The aim of this study was to examine user engagement with an app-based PA intervention. Use of the app was high, and although this trended downward, the low attrition rates indicate that most users were returning to the app at least once every 30 days throughout the intervention. Rates of use differed on the basis of demographic characteristics, where older users and those with a BMI in the healthy range used the app more. Unsurprisingly, engagement was higher for users of the gamified app, which included additional features designed to encourage this. Super users (top quartile of users) also tended to be older and were less likely to be obese. Super users increased their PA over time, whereas regular users decreased.

Engagement with the app was high compared with both research- and industry-led apps. In the gamified and basic app groups combined, users accessed the app features on an average of 175 (SD 165) times during the 100 days. In comparison, a systematic review reported rates of engagement ranging between 5 and 55 times (5%-15% of intended engagement) within Web-based health behavior interventions, ranging in duration from 3 months to 26 weeks [8]. Although engagement data from commercial apps are not readily available, a study of over a million users of a commercial weight loss app (Lose It!) that operationalized engagement as number of days of use found mean engagement of 29 days, which increased to 172 days for users accessing customizable features in the app (eg, personalized PA plan) [31]. Other reports examining all apps available in the Apple and Google Play app stores suggest that 21% of all downloaded apps are engaged with just once in the first 6 months [32]. Declining rates of engagement over time are often reported by researcher-led Web-based health interventions [8,9,33,34]; however, although our daily engagement dropped from a high of 70.8% (213/301) to a low of 13.3% (40/301), we found that most users (76.4%, 230/301) were still engaging with the app at least once every 30 days.

Rates of nonuse attrition in this study were low (<40%) compared with previous research and commercial apps. A similar RCT found that 80% of participants ceased using a Web-based PA intervention by week 80 [28], although this study duration is substantially longer than this study. In the Web-based 10,000 steps Australia study [29], 74.84% (8720/11,651) of app-only users had stopped using the intervention by day 43, although this higher rate is because the study occurred in a real world rather than controlled setting. Another real-world study of the 10,000 steps Australia website found that nonuse attrition occurred for 78% of participants after just 2 weeks [22]. Note that each of these studies [22,28,29] used 14 days of nonuse as their threshold, which is likely to have inflated their attrition rate compared with the 30-day threshold we used. When we applied the 14-day threshold in the sensitivity analysis, the attrition rate increased to 50% for the gamified group and 59% for the basic group across the 100-day period, which still compares favorably to the 10,000 steps studies [22,29]. However, our rate of attrition compared favorably with commercial apps, used in real-world settings, which experience an average rate of 62% [35] and which use the 30 consecutive days of nonuse threshold. That nonuse attrition was low in this study, and in that by Kolt et al [28], may be because of the fact that participants were using the app within the context of an RCT [36,37], compared with someone using an app under more ecologically valid circumstances. From our results, we cannot comment on engagement or attrition beyond the 100-day intervention period, as previous research reports increasingly rapid decline of usage as time passes [8,9,38-40].

Consistent with previous research [11], participant characteristics, namely being older and not being obese, were associated with increased engagement. Active Team was designed to be simple and easy to use, and it may have appealed to older users who are potentially less savvy in their use of technology or who may be more health conscious, as they tend to face more health problems [41-43]. Other intervention studies [29,44,45] have found higher engagement among older users, and a systematic review [11] reported a trend toward age and engagement being positively correlated. At first, the fact that users with higher BMI engaged with the app less is discouraging, and other studies have reported a similar negative association [11], as this suggests people who would benefit from the program the most were the least engaged, which is a common challenge for health promoters, whereby groups with the highest needs tend to be the hardest to recruit and engage [46,47]. However, in this study, a high percentage of overweight and obese users (78.4%, 236/301) compared with approximately 63% of the population) [48] were recruited; however, the engagement data show these users engaged less across the intervention period, perhaps as risks associated with PA, such as joint and respiratory problems [49-51], become more pronounced for people with higher BMIs, which does make engaging in PA more difficult. In contrast, an intervention using Fitbits found that overweight participants were more likely to engage and increase their PA [52], and more work should be undertaken to understand how best to engage different groups. Regardless, it should be noted that PA holds many health benefits beyond the control of body weight, including improved mood [53,54] and cardiorespiratory fitness [55], which means the higher engagement reported by participants who reported a BMI in the healthy or overweight range is still beneficial.

Users of the gamified app had higher rates of engagement (as measured by each of our metrics), and they were more likely to be super users (74%, 56/76 of super users had the gamified app). This suggests the social and gamified features enhanced engagement [56,57]. In this study, this is consistent with our intent at the outset, as the social and gamified features and prompts were specifically designed and included to increase engagement, these users also received push notifications when someone interacted with them in the app, which were intended to draw them back to the app. As all of our gamified features were social in nature, this network effect likely influenced rates of app use; use by 1 team member (or lack thereof) may have had a flow on effect, through the team, which either promoted or inhibited use. Although our current analysis does not allow us to comment on which social and gamified features resulted in increased engagement, our results suggest that future apps could benefit from including gamified features to promote comparison and competition among users. Moreover, some reviews [58,59] suggest the inclusion of more sophisticated gamified features (eg, personalized avatars, storylines) could enhance usage further. This should be considered with the caveat that there is ongoing debate around whether external motivation provided by gamification undermines intrinsic motivation for participating in PA [60-64]. This is particularly true for the gamified Active Team app, where continued usage may be dependent on usage by others, which could further undermine the intrinsic motivation. Although gamification does need to be implemented carefully to support establishment of and commitment to a PA regimen, the provision of an extrinsic motivator (to begin) is preferable to lacking any motivation.

Despite similar baseline PA levels, super users saw statistically significant increases in their objectively measured PA over time, when compared with regular users. These results held true after controlling for demographic characteristics likely to influence engagement (ie, age, BMI, sex, and education level [34]). Although promising, this does mean that regular users, who made up the majority of users (74.8%, (225/301), had PA levels that, on average, decreased slightly, 8 (SD 47) min, over the 3-month period. Taken together, these results suggest that app-based interventions can work, perhaps only for a small group of people or only when engagement can be maximized [8,65]. Although users of the gamified app had more features to engage with, these results are near identical when engagement was assessed using step calendar use, a feature that both groups had access to (see Multimedia Appendix 1). This suggests that self-monitoring of behavior may have been an engaging and potent enough mechanism for change for some users [66].

Together, these findings highlight the complexities of the theorized dose-response relationship between intervention exposure (engagement) and efficacy [8-10,65] and support the theory of an optimum dosage or exposure *threshold* [67] required for intervention efficacy, which is potentially different for each user. Given super users saw statistically significant increases to their PA over time, whereas those who used the app less did not, also suggests ongoing efforts to understand and boost engagement [12,13,68] are warranted to enhance appeal so that as many users as possible receive the optimum exposure for intervention benefits.

### Strengths, Limitations, and Future Directions

The findings presented here should be interpreted in the context of the study’s limitations. First, only user engagement during the 100-day intervention duration is included; we have relied on objective engagement metrics from the study’s server, and we have used the term gamification to refer to a particular set of game design elements (ie, newsfeed, challenges, virtual gifts, and leaderboard), all of which are social in nature. Furthermore, this study has not considered psychological aspects of engagement, such as participant perceptions or the interplay between personality and engagement [12,40]. In this study, super users were *a priori* defined as those whose app use fell within the top quartile of all users; however, we acknowledge that other definitions of this novel term would have been equally justified. Moreover, no single engagement metric can wholly capture and explain user engagement, as such, there will be limitations associated with any engagement metric. For example, the concept of nonuse attrition may be blunt, as it does fail to adequately capture usage that diminishes over time in those who continue to engage with the program. Our choice of engagement metric was guided by the metrics reported by industry-led apps, and it is important to note that such apps often view uptake and use as benchmark for success rather than behavior change. However, in research and public health settings, engagement is considered important, as it mediates user exposure to the intervention and therefore has important implications for efficacy. Causal claims cannot be made, as this study is a secondary and subgroup analysis of RCT data that did not experimentally test the impact of varying levels of engagement on PA outcomes. Reverse causation is equally possible; the relationships between app usage and PA may be because users who were able to increase their PA over time were more satisfied with their experience of the intervention; therefore, they used the app more. The sample is also subject to a self-selection bias and, typical of health behavior research, well-educated women are overrepresented [46].

Strengths of this study include the large sample size (n= 301) and the inclusion of objective measurements of app usage and PA. Although we acknowledge participants signing up to an RCT may be highly motivated, our intervention was delivered entirely via an app with no in-person contact between study staff and participants. However, the inclusion of eligibility criteria, baseline, and 3-month follow-up assessments signifies this approach is distinct from real-world settings, and we do not know whether our high rates of engagement are potentially influenced by a Hawthorne effect (where awareness of being under observation results in modified behavior [69,70]). Translation of researcher-led apps into real-world trials is likely to become more commonplace [7,21,22,71], and the ability to make comparisons to available industry-led apps will be increasingly important. This study has used engagement metrics commonly reported by industry-led apps, and the method and findings may be useful to future studies seeking to make direct comparisons between research- and industry-led apps.

A real-world trial of the app used in this study is now underway, and once complete, it can confirm whether the findings reported here hold true in a setting comparable to how we would expect people to use commercially available apps. Our findings support the inclusion of social and gamified features to increase engagement. Interventions are increasingly incorporating gamification and social features [45,72-75], and it will be interesting to see whether our engagement results are replicated in these studies. Moreover, although consensus on how engagement is operationalized has not yet eventuated, the field of engagement science can continue to progress if future studies publish analyses of app engagement that are similarly, or more, detailed (ie, to consider each gamified feature separately) than this study. These analyses should seek to evaluate the role of engagement levels on intervention efficacy in both instances where interventions are able to demonstrate overall efficacy and those that do not. In this way, engaging features can be identified, refined, and incorporated into future interventions to enhance their potential for positive behavior change effects.

### Conclusions

Taken together, our results indicate app-based interventions can sustain user engagement across a 100-day intervention period, and the inclusion of social and gamified features can enhance engagement. Users who were older or with a BMI classified as healthy or overweight (rather than obese) engaged more, and those who engaged the most (ie, super users, the top quartile of users) reported statistically significant increases to their objectively measured PA over time, supporting the theory that intervention exposure is linked to efficacy. Further research is now needed to determine whether these findings are replicated in other app- and Web-based behavior change studies and in ecologically valid real-world settings.

## Appendix

Multimedia Appendix 1 Step calendar use.

## Abbreviations

AAS: Active Australia Survey
BMI: body mass index
DAU: daily active user
MAU: monthly active user
MVPA: moderate-to-vigorous physical activity
PA: physical activity
RCT: randomized controlled trial
