# Supplementary material for: User Engagement and Attrition in an App-Based Physical Activity Intervention: Secondary Analysis of a Randomized Controlled Trial
Source: J Med Internet Res. 2019 Nov 27;21(11):e14645. doi: 10.2196/14645 (PMC6906621; doi:10.2196/14645)
Supplement: Multimedia Appendix 1 [file jmir_v21i11e14645_app1.pdf]

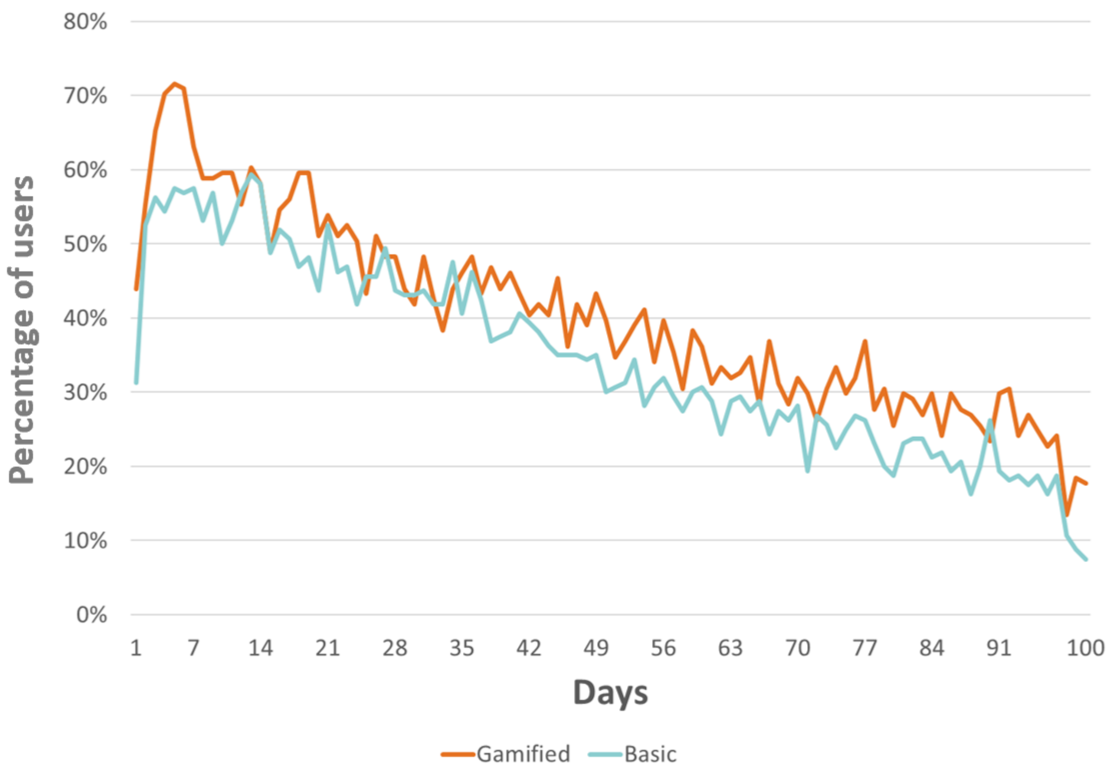

Figure 1 Daily active users - step calendar use

Churn

When assessed using the 30-days of non-use threshold, churn from step calendar use occurred in 35% and 40% of the gamified and basic groups, respectively, with no significant differences between groups in the time to churn (log-rank test, *p*= .30), see Figure 2. When the 14-days of non-use threshold was applied, churn occurred in 56% and 61% of the gamified and basic groups, respectively (log-rank test, *p*= .43), see Figure 3.

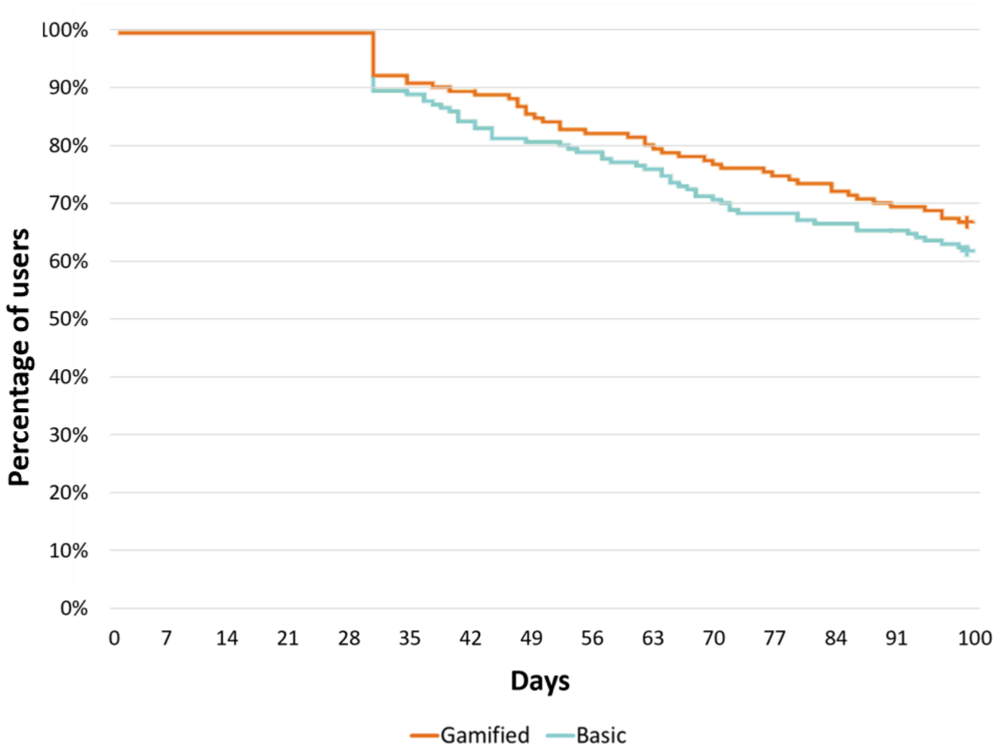

Figure 2 Kaplan-Meier survival estimates showing time to churn
30-day non-use threshold – step calendar use

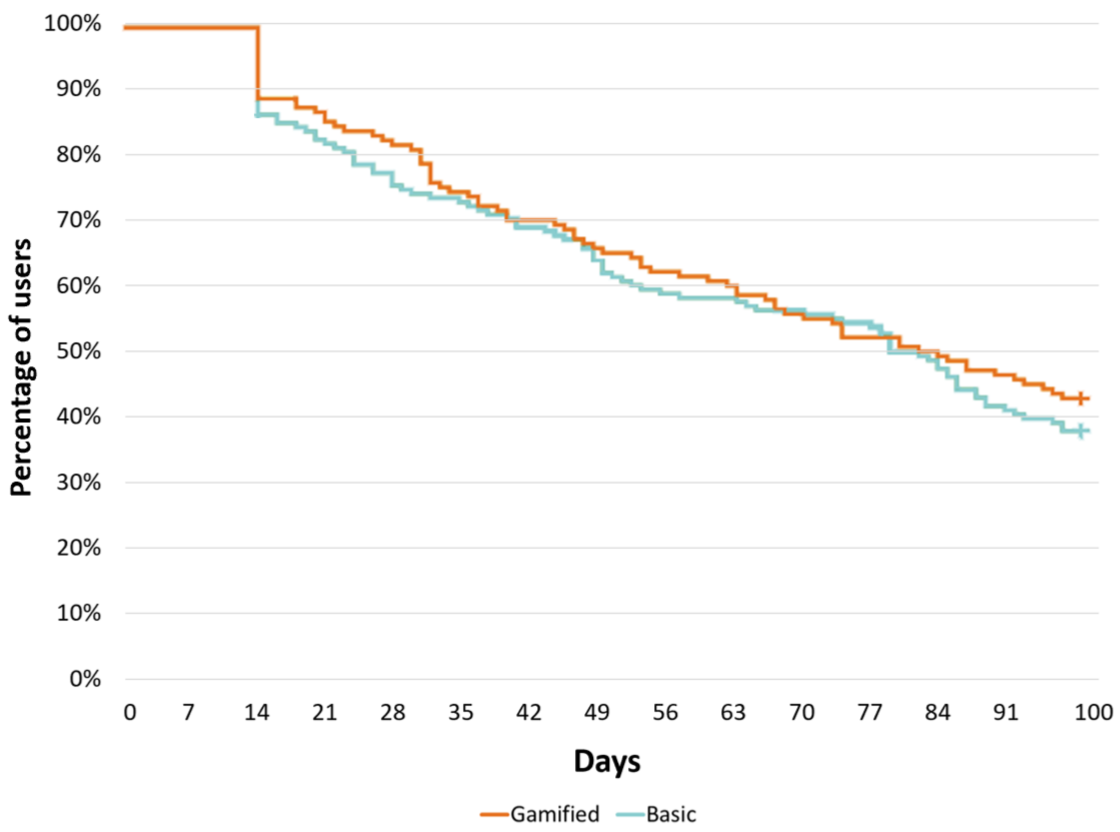

Figure 3 Kaplan-Meier survival estimates showing time to churn
14-day non-use threshold – step calendar use

Table 1 Associations between user baseline characteristics and step calendar use

			Step calendar use	
Characteristic	n	Mean (95% CI upper, lower)	*F*	*p*
Sex			1.22	.27
Male	78	38.7 (31.6, 45.8)		
Female	221	43.2 (38.6, 47.7)		
Education level			.93	.40
High School or less	51	41.2 (32.5, 49.8)		
Some college	89	38.0 (31.3, 44.8)		
University degree	159	43.6 (38.2, 49.0)		
BMI			8.99	**<.001**
Healthy weight	63	52.0 (43.8, 60.2)		
Overweight	107	39.1 (32.8, 45.5)		
Obese	129	31.6 (25.9, 37.4)		
Age			8.53	**.01**
Younger	149	35.6 (30.1, 41.2)		
Older	150	46.2 (40.4, 52.1)		
Group			4.68	**.03**
Gamified	141	44.8 (38.9, 50.6)		
Basic	160	37.1 (31.6, 42.5)		

Note: Age: younger = ≤40, older = ≥ 41. **Bold** indicates statistical significance.

Post hoc pairwise comparison (Kruskall-Wallis test) indicated total app use varied based on BMI ($\chi^2(2) = 13.36$, $p = .001$), driven by differences between those with a BMI classified as healthy (median total app use= 56), compared to obese (median total app use= 26)($p < .001$).

Step calendar use and changes to physical activity

There was a weak, significant total app use by time interaction effect for objective MVPA ($F = 7.6$, $p = .01$) and self-reported PA ($F = 4.4$, $p = .04$), where higher total app was associated with greater increases in PA by the three-month follow-up.

Super-users and changes to physical activity

At the three-month follow-up, objective MVPA had increased from baseline for super-users of the app while it decreased slightly for regular-users. There was a significant group by time interaction, where super-users were completing 28.2 (SE 9.5, 95% CI 9.4-46.9) more minutes of MVPA than regular-users ($F = 4.76$, $p = .03$). Differences between super- and regular-users for self-reported MVPA favoured the super-users (89.7, SE 43.4, 95% CI 4.4-175.1), yet did not reach statistical significance ($F = 3.31$, $p = .07$).

Table 2. Baseline characteristics and physical activity change scores comparisons between super- and regular-users, step calendar use

	Step calendar use		
	Super	Regular	*p*
Sex, n (%)			.16
Male	15 (20.0)	64 (28.3)	
Female	60 (80.0)	162 (71.7)	
Education level, n (%)			.02
High school or less	12 (16.2)	39 (17.3)	
Some college	13 (17.6)	76 (33.8)	
University degree	49 (66.2)	110 (48.9)	
BMI, n (%)			<.001
Healthy weight	26 (35.1)	37 (16.4)	
Overweight	28 (37.8)	79 (35.1)	
Obese	20 (27.0)	109 (48.4)	
Age, m (SD)	44.7 (12.1)	40.8 (11.8)	.02
Group, n (%)			.01
Gamified	45 (60.0)	96 (42.5)	
Basic	30 (40.0)	130 (57.5)	
Objective PA min/d - baseline, m (SD)	112.1 (48.8)	102.1 (52.4)	.14
Self-report PA min/wk - baseline, m (SD)	225.1 (203.7)	262.9 (251.7)	.24
Objective PA min/d change, m (SD)	19.9 (42.2)	-8.4 (47.9)	<.001
Self-report min/wk PA change, m (SD)	250.6 (435.7)	153.4 (331.8)	.09
Total app use, m (SD)	386.3 (172.3)	106.0 (83.4)	
Step calendar use, m (SD)	83.6 (10.5)	25.1 (18.8)	

m= mean, SD= standard deviation, min= minute, d= day, wk= week; PA physical activity change is from baseline to 3-month follow-up (end of intervention).
